# Activities of Manogepix and Comparators against 1,435 Recent Fungal Isolates Collected during an International Surveillance Program (2020)

**DOI:** 10.1128/aac.01028-22

**Published:** 2022-10-26

**Authors:** M. A. Pfaller, M. D. Huband, P. R. Rhomberg, P. A. Bien, M. Castanheira

**Affiliations:** a JMI Laboratories, North Liberty, Iowa, USA; b University of Iowa, Iowa City, Iowa, USA; c Pfizer Inc., New York, New York, USA

**Keywords:** APX001, APX001A, Gwt1, antifungal, fosmanogepix, manogepix

## Abstract

We evaluated the *in vitro* activity of manogepix and comparator agents against 1,435 contemporary fungal isolates collected worldwide from 73 medical centers in North America, Europe, the Asia-Pacific region, and Latin America during 2020. Of the isolates tested, 74.7% were *Candida* spp.; 3.7% were non-*Candida* yeasts, including 27 Cryptococcus neoformans var. *grubii* (1.9%); 17.1% were Aspergillus spp.; and 4.5% were other molds. All fungal isolates were tested by reference broth microdilution according to CLSI methods. Based on MIC_90_ values, manogepix (MIC_50_/MIC_90_, 0.008/0.06 mg/liter) was 16- to 64-fold more active than anidulafungin, micafungin, and fluconazole against *Candida* spp. isolates and the most active agent tested. Similarly, manogepix (MIC_50_/MIC_90_, 0.5/1 mg/liter) was ≥8-fold more active than anidulafungin, micafungin, and fluconazole against C. neoformans var. *grubii*. Based on minimum effective concentration for 90% of the isolates tested (MEC_90_) and MIC_90_ values, manogepix (MEC_90_, 0.03 mg/liter) was 16- to 64-fold more potent than itraconazole, posaconazole, and voriconazole (MIC_90_s, 0.5 to 2 mg/liter) against 246 Aspergillus spp. isolates. Aspergillus fumigatus isolates exhibited a wild-type (WT) phenotype for the mold-active triazoles, including itraconazole (87.0% WT) and voriconazole (96.4% WT). Manogepix was highly active against uncommon species of *Candida*, non-*Candida* yeasts, and rare molds, including 11 isolates of Candida auris (MIC_50_/MIC_90_, 0.004/0.015 mg/liter) and 12 isolates of *Scedosporium* spp. (MEC_50_/MEC_90_, 0.06/0.12 mg/liter). Additional studies are in progress to evaluate the clinical utility of the manogepix prodrug fosmanogepix in difficult-to-treat resistant fungal infections.

## INTRODUCTION

Invasive fungal infections (IFI) due to opportunistic fungal pathogens pose a major stumbling block to the successful implementation of advances in medical therapy (1, 2). Whereas the majority of IFI and associated deaths are due to Aspergillus, *Candida*, and Cryptococcus species, other less common opportunistic yeasts and molds increasingly are emerging as deadly antifungal resistant pathogens (1–6). Accordingly, new antifungal therapies that act through novel mechanisms of action are needed to control the high mortality of IFIs and combat the emergence of resistance to existing treatment regimens. Several antifungal agents with the potential to address the emergence of multidrug-resistant yeasts and molds (i.e., resistant to at least 2 different classes of antifungal agents) are presently in clinical development (7–13).

Among the more recent, systemically active antifungal agents, manogepix (formerly APX001A and E1210) is notable for its unique mechanism of action. Manogepix targets the highly conserved fungal enzyme Gwt1 (14). Inhibiting Gwt1 blocks the inositol acylation step during the synthesis of glycosylphosphatidylinositol-anchored proteins of the fungal cell wall. This inhibition compromises cell wall integrity, biofilm formation, and germ tube formation, resulting in severe fungal growth defects (14). Manogepix demonstrates broad-spectrum antifungal activity against common species of *Candida*, Cryptococcus neoformans, Cryptococcus gattii, Aspergillus spp., multidrug-resistant strains such as Candida auris, and rare molds, which are often difficult to treat due to their inherent resistance to most antifungals, including Fusarium spp., *Scedosporium* spp., and *Lomentospora* (*Scedosporium*) *prolificans* (12, 13, 15–22).

In the present study, we utilized SENTRY Antimicrobial Surveillance Program data from 2020 to examine the *in vitro* activity of manogepix as well as its comparators, anidulafungin, micafungin, fluconazole, itraconazole, posaconazole, voriconazole, and amphotericin B, against 1,435 contemporary clinical fungal isolates from bloodstream infections (BSIs), respiratory tract infections (RTIs), skin and skin structure infections (SSSIs), urinary tract infections (UTIs), intra-abdominal infections (IAIs), and other infection types. The fungal isolates were collected from 73 medical centers located in North America (568 isolates from 29 medical centers), Europe (566 isolates from 28 medical centers), the Asia-Pacific region (182 isolates from 10 medical centers), and Latin America (119 isolates from 6 medical centers). These data expand on our previous reports from 2017 (20) and 2018–2019 (12), allowing examination of temporal trends in fungal species distribution and antifungal susceptibility results as well as providing a robust MIC database for eventual determination of both epidemiological cutoff values (ECVs) and clinical breakpoints (CBPs) for manogepix and other antifungal agents and a variety of fungal species.

## RESULTS

The frequency distributions and cumulative percent inhibition data for manogepix against the species and organism groups tested are listed in Table 1. All fungal species containing ≥10 isolates were analyzed separately. Manogepix and comparator agent susceptibility results for fungal species with fewer than 10 isolates are listed in Tables S1 and S2 in the supplemental material.

**TABLE 1:** Manogepix frequency and cumulative percent inhibition against the main organisms and organism groups tested using the CLSI broth microdilution method

Organism group or organism (no. of isolates)	No. (cumulative %) of isolates inhibited at MIC or MEC (mg/liter) of^*a*^:														MIC_50_	MIC_90_
	≤0.002	0.004	0.008	0.015	0.03	0.06	0.12	0.25	0.5	1	2	4	8	>^*b*^		
*Candida* spp. (1,072)	29 (2.7)	226 (23.8)	**313** (**53.0**)	218 (73.3)	150 (87.3)	69 (93.8)	13 (95.0)	6 (95.5)	2 (95.7)	2 (95.9)	1 (96.0)	1 (96.1)	5 (96.5)	37 (100.0)	0.008	0.06
C. albicans (350)	12 (3.4)	**178** (**54.3**)	152 (97.7)	6 (99.4)	1 (99.7)	0 (99.7)	1 (100.0)								0.004	0.008
C. auris (11)	3 (27.3)	**6** (**81.8**)	0 (81.8)	1 (90.9)	1 (100.0)										0.004	0.015
C. dubliniensis (40)	5 (12.5)	**25** (**75.0**)	10 (100.0)												0.004	0.008
C. glabrata (258)	0 (0.0)	1 (0.4)	20 (8.1)	54 (29.1)	**112** (**72.5**)	62 (96.5)	9 (100.0)								0.03	0.06
C. kefyr (10)					0 (0.0)	1 (10.0)	2 (30.0)	**4** (**70.0**)	1 (80.0)	2 (100.0)					0.25	1
C. krusei (45)							0 (0.0)	2 (4.4)	0 (4.4)	0 (4.4)	0 (4.4)	1 (6.7)	5 (17.8)	**37** (**100.0**)	>8	>8
C. lusitaniae (16)			0 (0.0)	5 (31.2)	**7** (**75.0**)	4 (100.0)									0.03	0.06
C. orthopsilosis (12)		0 (0.0)	**7** (**58.3**)	3 (83.3)	2 (100.0)										0.008	0.03
C. parapsilosis (165)	0 (0.0)	4 (2.4)	89 (56.4)	58 (91.5)	13 (99.4)	1 (100.0)									0.008	0.015
C. tropicalis (139)	1 (0.7)	6 (5.0)	30 (26.6)	**89 (90.6)**	11 (98.6)	1 (99.3)	1 (100.0)								0.015	0.015
Other *Candida* spp. (24)^*c*^	**8** (**33.3**)	5 (54.2)	4 (70.8)	2 (79.2)	3 (91.7)	0 (91.7)	0 (91.7)	0 (91.7)	1 (95.8)	0 (95.8)	1 (100.0)				0.004	0.03
Cryptococcus neoformans var. *grubii* (27)				0 (0.0)	2 (7.4)	5 (25.9)	3 (37.0)	2 (44.4)	**8** (**74.1**)	7 (100.0)					0.5	1
Other Yeasts (26)^*d*^		0 (0.0)	3 (11.5)	5 (30.8)	**7** (**57.7**)	3 (69.2)	1 (73.1)	1 (76.9)	1 (80.8)	1 (84.6)	0 (84.6)	0 (84.6)	0 (84.6)	4 (100.0)	0.03	>8
Aspergillus spp. (246)	0 (0.0)	7 (2.8)	47 (22.0)	**141** (**79.3**)	45 (97.6)	6 (100.0)									0.015	0.03
A. fumigatus (169)		0 (0.0)	20 (11.8)	**110** (**76.9**)	36 (98.2)	3 (100.0)									0.015	0.03
Aspergillus section Flavi (24)		0 (0.0)	3 (12.5)	**13** (**66.7**)	5 (87.5)	3 (100.0)									0.015	0.06
Aspergillus section Nigri (27)	0 (0.0)	6 (22.2)	**14** (**74.1**)	5 (92.6)	2 (100.0)										0.008	0.015
Aspergillus section Terrei (14)	0 (0.0)	1 (7.1)	**7** (**57.1**)	5 (92.9)	1 (100.0)										0.008	0.015
Other Aspergillus spp. (12)^*e*^		0 (0.0)	3 (25.0)	**8** (**91.7**)	1 (100.0)										0.015	0.015
*Scedosporium* spp. (12)^*f*^				0 (0.0)	3 (25.0)	**7** (**83.3**)	1 (91.7)	0 (91.7)	1 (100.0)						0.06	0.12
Other molds (52)^*g*^	6 (11.5)	9 (28.8)	**11** (**50.0**)	8 (65.4)	2 (69.2)	3 (75.0)	0 (75.0)	2 (78.8)	0 (78.8)	2 (82.7)	2 (86.5)	3 (92.3)	1 (94.2)	3 (100.0)	0.008	4

a The 24-h MIC recorded for *Candida* spp., 48-h MIC recorded for other yeasts and C. neoformans var. *grubii*; 72-h MEC recorded for *Scedosporium* spp., or 48-h MEC recorded for Aspergillus spp. and other molds. Numbers in boldface are modal MIC values.

b Greater than the highest dilution tested.

c Organisms (number of isolates) include *Candida bracarensis* (2), *C. duobushaemulonii* (1), *C. fermentati* (2), C. guilliermondii (6), *C. haemulonii* (1), C. inconspicua (2), C. nivariensis (1), C. pelliculosa (4), *C. rugosa* (2), *C. spencermartinsiae* (1), and *C. utilis* (2).

d Organisms (number of isolates) include Cryptococcus gattii *species complex* (1), C. neoformans var. *neoformans* (2), *Saprochaete clavata* (3), *Kodamaea ohmeri* (1), Magnusiomyces capitatus (1), Rhodotorula mucilaginosa (5), Saccharomyces cerevisiae (7), *Trichosporon asahii* (4), *T. mycotoxinivorans* (1), and Yarrowia lipolytica (1).

e Organisms (number of isolates) include Aspergillus nidulans (6), A. nidulans species complex (2), *A. sclerotiorum* (1), A. ustus (1), A. ustus species complex (1), and A. versicolor (1).

f Organisms (number of isolates) include Scedosporium apiospermum*/*Scedosporium boydii (9) and S. aurantiacum (3).

g Organisms (number of isolates) include *Exophiala dermatitidis* (1), Fusarium *incarnatum-equiseti* species complex (1), F. solani (2), F. solani species complex (5), *Gibberella fujikuroi* species complex (6), Lichtheimia corymbifera (1), *Lomentospora prolificans* (4), Mucor circinelloides (2), *M. indicus* (1), *Purpuriocillium lilacinum* (2), P. variotii (7), Penicillium citrinum (1), *P. onobense* (1), *Rasamsonia argillacea* (1), *R. argillacea* species complex (3), Rhizopus microsporus group (4), *R. oryzae* (1), *R. oryzae* species complex (1), *Sarocladium kiliense* (1), *Scopulariopsis brevicaulis* (1), unspeciated *Acremonium* (1), unspeciated *Coprinellus* (1), unspeciated *Cunninghamella* (1), unspeciated *Lichtheimia* (1), unspeciated *Paecilomyces* (1), and unspeciated *Trichoderma* (1).

Of the 1,435 fungal clinical isolates tested, 1,072 (74.7%) were *Candida* spp.; 53 (3.7%) were non-*Candida* yeasts, including 27 Cryptococcus neoformans var. *grubii* (1.9%); 246 (17.1%) were Aspergillus spp.; and 64 (4.5%) were other molds (Table 1; Tables S1 and S2). The geographic distribution of fungal isolates was 39.6% from North America, 39.4% from Europe, 12.7% from the Asia-Pacific region, and 8.3% from Latin America (data not shown).

### Activity of manogepix against *Candida* spp. and Cryptococcus neoformans var. *grubii* isolates.

Among the 10 species of *Candida* in Table 1, manogepix was most active against Candida albicans and Candida dubliniensis (MIC_90_, 0.008 mg/liter) and least active against Candida kefyr (MIC_90_, 1 mg/liter) and Candida krusei (MIC_90_, >8 mg/liter). C. krusei (MIC_50_/MIC_90_, >8/>8 mg/liter) is considered intrinsically resistant to manogepix (13). All C. auris isolates (*n *= 11) were inhibited by ≤0.03 mg/liter manogepix. Overall, 93.8% of the 1,072 *Candida* spp. isolates tested were inhibited by ≤0.06 mg/liter manogepix and 95.5% were inhibited by ≤0.25 mg/liter manogepix (Table 1). Manogepix had an MIC distribution spanning six 2-fold dilution steps (range, 0.03 to 1 mg/liter) and did not have a clear mode against 27 C. neoformans var. *grubii* isolates (MIC_50_/MIC_90_, 0.5/1 mg/liter; 100.0% inhibited at ≤1 mg/liter) (Table 1).

### Determination of the wild-type manogepix MIC distribution for *Candida* spp.

The upper limit of the manogepix wild-type MIC distribution (WT-UL, two 2-fold dilutions higher than the modal MIC value) for each species was determined by compiling the data from the 2017 (20), 2018–2019 (12), and 2020 (present study) SENTRY Surveillance Program surveys to provide a robust set of values representing the WT MIC distributions as determined by CLSI methods (Table 2). Importantly, the modal manogepix MIC value for each species was within 1 dilution step, as were the MIC_50_/MIC_90_ values, irrespective of the individual survey. The similarity of these values ensured comparable MIC distributions across the three surveys. The WT-UL cutoff value was determined for each species by using the combined 2017 to 2020 MIC distribution (Table 2).

**TABLE 2 T2:** Summary of manogepix surveillance data as determined by CLSI broth microdilution methods for *Candida* spp. and Aspergillus spp. in this and prior studies

Organism	Yr(s)	*n*	MIC_50_/MIC_90_ (mg/liter)	Mode (mg/liter)	WT-UL (mg/liter)	Reference
C. albicans	2017	414	0.008/0.008	0.008	0.03 (100.0%)^*a*^	Pfaller et al. (20)
	2018–2019	588	0.004/0.008	0.004/0.008^*b*^	0.03 (100.0%)	Pfaller et al. (12)
	2020	350	0.004/0.008	0.004/0.008^*b*^	0.03 (99.7%)	This study
	2017–2020	1,352	0.004/0.008	0.004/0.008^*b*^	0.03 (99.9%)	Overall
C. glabrata	2017	321	0.06/0.12	0.06	0.25 (100.0%)	Pfaller et al. (20)
	2018–2019	460	0.03/0.06	0.03	0.12 (100.0%)	Pfaller et al. (12)
	2020	258	0.03/0.06	0.03	0.12 (100.0%)	This study
	2017–2020	1,039	0.03/0.06	0.03/0.06^*b*^	0.25 (100.0%)	Overall
C. parapsilosis	2017	270	0.008/0.015	0.008	0.03 (98.9%)	Pfaller et al. (20)
	2018–2019	321	0.008/0.015	0.008	0.03 (98.4%)	Pfaller et al. (12)
	2020	165	0.008/0.015	0.008	0.03 (99.4%)	This study
	2017–2020	756	0.008/0.015	0.008	0.03 (98.8%)	Overall
C. tropicalis	2017	151	0.015/0.03	0.015	0.06 (100.0%)	Pfaller et al. (20)
	2018–2019	225	0.015/0.015	0.008/0.015^*b*^	0.06 (99.6%)	Pfaller et al. (12)
	2020	139	0.015/0.015	0.015	0.06 (99.3%)	This study
	2017–2020	515	0.015/0.03	0.015	0.06 (99.6%)	Overall
C. dubliniensis	2017	49	0.004/0.008	0.004	0.015 (100.0%)	Pfaller et al. (20)
	2018–2019	65	0.004/0.008	0.004	0.015 (98.5%)	Pfaller et al. (12)
	2020	40	0.004/0.008	0.004	0.008 (100.0%)	This study
	2017–2020	154	0.004/0.008	0.004	0.015 (99.4%)	Overall
C. lusitaniae	2017	39	0.03/0.12	NM^*c*^	NA^*d*^	Pfaller et al. (20)
	2018–2019	52	0.03/0.06	0.03	0.12 (98.1%)	Pfaller et al. (12)
	2020	16	0.03/0.06	0.03	0.06 (100.0%)	This study
	2017–2020	107	0.03/0.12	0.03	0.12 (97.2%)	Overall
C. kefyr	2017	13	0.12/0.5	0.06/0.12^*b*^	0.5 (100.0%)	Pfaller et al. (20)
	2018–2019	28	0.12/0.25	0.12/0.25^*b*^	0.5 (100.0%)	Pfaller et al. (12)
	2020	10	0.25/1	0.25	1 (100.0%)	This study
	2017–2020	51	0.12/0.5	0.12/0.25^*b*^	1 (100.0%)	Overall
A. fumigatus	2017	182	0.015/0.03	0.015	0.06 (100.0%)	Pfaller et al. (20)
	2018–2019	397	0.015/0.03	0.015	0.06 (100.0%)	Pfaller et al. (12)
	2020	169	0.015/0.03	0.015	0.06 (100.0%)	This study
	2017–2020	748	0.015/0.03	0.015	0.06 (100.0%)	Overall
Aspergillus section Flavi	2017	18	0.015/0.03	0.03	0.06 (100.0%)	Pfaller et al. (20)
	2018–2019	73	0.015/0.03	0.015	0.06 (100.0%)	Pfaller et al. (12)
	2020	24	0.015/0.06	0.015	0.06 (100.0%)	This study
	2017–2020	115	0.015/0.03	0.015	0.06 (100.0%)	Overall
Aspergillus section Nigri	2017	23	≤0.008/0.015	≤0.008	0.03 (100.0%)	Pfaller et al. (20)
	2018–2019	67	0.008/0.015	0.015	0.03 (100.0%)	Pfaller et al. (12)
	2020	27	0.008/0.015	0.008	0.03 (100.0%)	This study
	2017–2020	117	≤0.008/0.015	≤0.008	0.03 (100.0%)	Overall
Aspergillus section Terrei	2017	10	0.015/0.03	0.015	0.03 (100.0%)	Pfaller et al. (20)
	2018–2019	19	0.008/0.03	0.008	0.03 (100.0%)	Pfaller et al. (12)
	2020	14	0.008/0.015	0.008	0.03 (100.0%)	This study
	2017–2020	43	0.015/0.03	0.015	0.03 (100.0%)	Overall

a Percent of isolates encompassed by WT-UL.

b Bimodal MIC distribution.

c NM, no mode.

d NA, not applicable.

The upper limit of the WT MIC distribution for manogepix was 0.015 mg/liter for C. dubliniensis (99.4% WT; 153/154 isolates), 0.03 mg/liter for C. albicans (99.9% WT; 1,351/1,352 isolates), 0.03 mg/liter for Candida parapsilosis (98.8% WT; 747/756 isolates), 0.06 mg/liter for Candida tropicalis (99.6% WT; 513/515 isolates), 0.25 mg/liter for Candida glabrata (100.0% WT; 1,039/1,039 isolates), 0.12 mg/liter for Candida lusitaniae (97.2% WT; 104/107 isolates), and 1 mg/liter for C. kefyr (100.0% WT; 51/51 isolates) (Table 2). The WT-UL MIC for 23 C. auris isolates could not be determined due to the lack of a clear mode (data not shown).

### *In vitro* activity of manogepix and comparators against *Candida* spp. and Cryptococcus neoformans var. *grubii* isolates.

Of the 350 C. albicans isolates tested, all but 1 were inhibited by ≤0.03 mg/liter manogepix (99.7% WT; MIC_50_/MIC_90_, 0.004/0.008 mg/liter), and echinocandin susceptibility was 99.7% for micafungin and 100.0% for anidulafungin using current CLSI M27M44S (23) breakpoint interpretive criteria (Tables 1 and 3). All but 3 C. albicans isolates were susceptible to fluconazole (99.1%), 99.7% were susceptible to voriconazole, and 97.4% were WT (MIC, ≤0.06 mg/liter) to posaconazole (Table 3). A single C. albicans isolate for which the echinocandin MIC values were greater than the ECV was screened for the presence of *fks* hot spot (HS) mutations (Table 4). This isolate from Taiwan displayed amino acid alteration *fks1* HS1 S645P (an S-to-P change at position 645) (Table 4). Its corresponding manogepix MIC value was 0.008 mg/liter (Table 4).

**TABLE 3:** *In vitro* activity of manogepix and comparator agents tested against *Candida* spp. and C. neoformans isolates

Organism (no. tested) and antifungal agent	MIC data (mg/liter)			CLSI CBP^*a*^			CLSI ECV^*a*^	
	MIC_50_	MIC_90_	Range	% S	% I/SDD	% R	% WT	% NWT
C. albicans (350)								
Manogepix	0.004	0.008	≤0.002–0.12				99.7	0.3
Anidulafungin	0.03	0.06	≤0.002–0.25	100.0	0.0	0.0	99.7	0.3
Micafungin	0.015	0.015	≤0.002–2	99.7	0.0	0.3	99.1	0.9
Fluconazole	0.12	0.25	≤0.008–>128	99.1	0.3	0.6^*b*^	97.1	2.9
Posaconazole	0.03	0.06	0.004–>8				97.4	2.6
Voriconazole	0.004	0.015	≤0.002–>8	99.7	0.0	0.3	98.6	1.4
Amphotericin B	0.5	0.5	0.06–1				100.0	0.0
C. glabrata (258)								
Manogepix	0.03	0.06	0.004–0.12				100.0	0.0
Anidulafungin	0.12	0.12	0.03–4	96.5	1.2	2.3	97.7	2.3
Micafungin	0.015	0.03	0.008–4	97.3	0.0	2.7	96.9	3.1
Fluconazole	4	8	0.12–>128		95.0	5.0^*c*^	90.3	9.7
Posaconazole	0.5	1	0.12–>8				96.1	3.9
Voriconazole	0.12	0.25	0.015–8				90.7	9.3
Amphotericin B	1	1	0.25–1				100.0	0.0
C. parapsilosis (165)								
Manogepix	0.008	0.015	0.004–0.06				99.4	0.6
Anidulafungin	2	2	0.5–4	92.1	7.9	0.0	100.0	0.0
Micafungin	1	1	0.25–2	100.0	0.0	0.0	100.0	0.0
Fluconazole	0.5	2	0.12–128	92.1	0.0	7.9^*b*^	92.1	7.9
Posaconazole	0.06	0.12	0.015–0.25				100.0	0.0
Voriconazole	0.008	0.03	≤0.002–1	94.5	3.6	1.8	91.5	8.5
Amphotericin B	0.5	1	0.25–1				100.0	0.0
C. tropicalis (139)								
Manogepix	0.015	0.015	≤0.002–0.12				99.3	0.7
Anidulafungin	0.03	0.06	0.008–0.25	100.0	0.0	0.0	100.0	0.0
Micafungin	0.03	0.03	0.008–0.06	100.0	0.0	0.0	100.0	0.0
Fluconazole	0.5	1	0.06–>128	95.0	1.4	3.6^*b*^	94.2	5.8
Posaconazole	0.06	0.12	0.015–>8				94.2	5.8
Voriconazole	0.03	0.06	0.004–>8	95.7	1.4	2.9	95.7	4.3
Amphotericin B	0.5	1	0.25–1				100.0	0.0
C. auris (11)								
Manogepix	0.004	0.015	≤0.002–0.03					
Anidulafungin	0.25	0.25	0.12–0.25	100.0		0.0^*d*^		
Micafungin	0.12	0.12	0.06–0.12	100.0		0.0^*d*^		
Fluconazole	32	>128	2–>128	36.4		63.6^*d*^		
Posaconazole	0.06	0.5	0.03–0.5					
Voriconazole	0.12	1	0.015–1					
Amphotericin B	1	2	1–2	72.7		27.3^*d*^		
C. dubliniensis (40)								
Manogepix	0.004	0.008	≤0.002–0.008				100.0	0.0
Anidulafungin	0.06	0.12	0.015–0.12				100.0	0.0
Micafungin	0.015	0.03	0.008–0.06				100.0	0.0
Fluconazole	0.12	0.25	0.06–0.25				100.0	0.0
Posaconazole	0.03	0.06	0.015–0.06				100.0	0.0
Voriconazole	0.004	0.008	≤0.002–0.015					
Amphotericin B	0.25	0.5	0.12–1				97.5	2.5
C. kefyr (10)								
Manogepix	0.25	1	0.06–1				100.0	0.0
Anidulafungin	0.06	0.12	0.06–0.25				100.0	0.0
Micafungin	0.06	0.12	0.03–0.12				100.0	0.0
Fluconazole	0.5	0.5	0.06–0.5				100.0	0.0
Posaconazole	0.12	0.12	0.06–0.25				100.0	0.0
Voriconazole	0.008	0.008	≤0.002–0.015					
Amphotericin B	1	1	0.5–1				100.0	0.0
C. lusitaniae (16)								
Manogepix	0.03	0.06	0.015–0.06				100.0	0.0
Anidulafungin	0.5	1	0.12–1				100.0	0.0
Micafungin	0.12	0.25	0.06–0.25				100.0	0.0
Fluconazole	0.5	1	0.12–4				93.8	6.2
Posaconazole	0.06	0.12	0.03–0.12				87.5	12.5
Voriconazole	0.008	0.015	0.004–0.03					
Amphotericin B	0.5	0.5	0.25–1				100.0	0.0^*e*^
C. orthopsilosis (12)								
Manogepix	0.008	0.03	0.008–0.03					
Anidulafungin	0.5	1	0.25–1				100.0	0.0
Micafungin	0.25	0.5	0.12–0.5				100.0	0.0
Fluconazole	0.5	128	0.5–>128				83.3	16.7
Posaconazole	0.06	0.5	0.06–0.5				83.3	16.7
Voriconazole	0.015	4	0.008–8				83.3	16.7
Amphotericin B	0.5	0.5	0.25–0.5				100.0	0.0
Cryptococcus neoformans var. *grubii* (27)								
Manogepix	0.5	1	0.03–1					
Anidulafungin	>4	>4	>4–>4					
Micafungin	>4	>4	>4–>4					
Fluconazole	4	8	0.5–8				100.0	0.0
Posaconazole	0.12	0.25	0.03–0.25				100.0	0.0
Voriconazole	0.06	0.06	0.008–0.12				100.0	0.0
Amphotericin B	0.5	1	0.5–1				51.9	48.1

a Clinical breakpoint (CBP) MIC criteria were those published in CLSI document M27M44S (23) and M38M51S (34). ECV criteria were those published in CLSI document M57S (24). The WT-UL was used in place of ECV for manogepix (see Table 2).

b Intermediate was interpreted as susceptible/dose dependent.

c Nonresistant was interpreted as susceptible/dose dependent.

d Breakpoints for this organism originated from the CDC tentative MIC breakpoints published at https://www.cdc.gov/fungal/candida-auris/c-auris-antifungal.html.

e Candida lusitaniae is not intrinsically resistant to amphotericin B. However, C. lusitaniae may develop resistance to amphotericin B *in vivo* during therapy.

**TABLE 4:** Summary of FKS alterations detected in *Candida* sp. isolates as part of the 2020 international surveillance program

Country	Organism	MIC (mg/liter)^*a*^			1,3-β-D-glucan synthase alteration			
		Manogepix	Anidulafungin	Micafungin	*fks1* HS1	*fks1* HS2	*fks2* HS1	*fks2* HS2
Taiwan	C. albicans	0.008	0.25 (S)	2 (R)	S645P	WT^*b*^	NT^*c*^	NT
USA	C. glabrata	0.12	1 (R)	0.25 (R)	D632E	WT	WT	WT
USA	C. glabrata	0.008	0.5 (R)	0.25 (R)	WT	WT	R665G	WT
USA	C. glabrata	0.03	2 (R)	1 (R)	S629P	WT	WT	WT
USA	C. glabrata	0.03	1 (R)	0.5 (R)	WT	WT	S663P	WT
USA	C. glabrata	0.06	0.25 (I)	0.5 (R)	WT	WT	WT	WT
USA	C. glabrata	0.06	4 (R)	4 (R)	WT	WT	S663P	WT
USA	C. glabrata	0.06	2 (R)	0.5 (R)	WT	WT	S663P	WT

a Determined according to the CLSI method. Categorical interpretations of susceptible (S), intermediate (I), and resistant (R) followed CLSI breakpoints (CLSI document M27M44S, 2022 [23]).

b WT, wild type.

c NT, Not tested.

All (100%) of the 258 C. glabrata isolates tested were inhibited by manogepix (MIC_50_/MIC_90_, 0.03/0.06 mg/liter) at the WT-UL MIC cutoff value of ≤0.25 mg/liter (Tables 1 [Table T2]to 3). Micafungin (MIC_50_/MIC_90_, 0.015/0.03 mg/liter) and anidulafungin (MIC_50_/MIC_90_, 0.12/0.12 mg/liter) susceptibilities were 97.3% and 96.5%, respectively, at the current CLSI breakpoints for these compounds (23). Seven C. glabrata isolates displayed echinocandin MIC values greater than the CLSI ECV and were screened for the presence of *fks* HS mutations. Of these isolates, 6 harbored amino acid alterations (Table 4). The most common substitution was *fks2* HS1 S663P (3 isolates). Two isolates carried mutations in *fks1* (HS1; D632E or S629P), and one carried a mutation in *fks2* (HS1; R665G). The 6 echinocandin nonsusceptible isolates with *fks* mutations, all of which were resistant (R) to micafungin, were from the United States and represented 5.3% of North American C. glabrata isolates (Table 4). Manogepix MIC values against these echinocandin-R C. glabrata isolates ranged from 0.008 to 0.12 mg/liter (all ≤WT-UL) (Table 4). Resistance of C. glabrata isolates to fluconazole was 5.0% (Table 3). A total of 3.9% and 9.3% of C. glabrata isolates were non-wild type (NWT) to posaconazole and voriconazole, respectively, using the ECVs published by CLSI (24) (Table 3).

Manogepix (MIC_50_/MIC_90_, 0.008/0.015 mg/liter) inhibited 99.4% of 165 C. parapsilosis isolates at the WT-UL of ≤0.03 mg/liter (Tables 1 [Table T2]to 3). Micafungin (MIC_50_/MIC_90_, 1/1 mg/liter) and anidulafungin (MIC_50_/MIC_90_, 2/2 mg/liter) susceptibilities were 100.0% and 92.1%, respectively, at the current CLSI C. parapsilosis breakpoints for these compounds (Table 3). A total of 7.9% of C. parapsilosis isolates were intermediate in susceptibility to anidulafungin (MIC, 4 mg/liter) (Table 3). Susceptibility of C. parapsilosis isolates to fluconazole and voriconazole was 92.1% and 94.5%, respectively, using current CLSI breakpoint interpretive criteria (Table 3). All (100.0%) C. parapsilosis isolates were WT to posaconazole (Table 3). The other member of the C. parapsilosis species complex (SC), Candida orthopsilosis, tended to be slightly more susceptible than C. parapsilosis *sensu stricto* to the echinocandins and was equally susceptible to manogepix and less susceptible to the azoles (Table 3).

Manogepix (MIC_50_/MIC_90_, 0.015/0.015 mg/liter), anidulafungin (MIC_50_/MIC_90_, 0.03/0.06 mg/liter; 100.0% susceptible), and micafungin (MIC_50_/MIC_90_, 0.03/0.03 mg/liter; 100.0% susceptible) displayed comparable activities against 139 C. tropicalis isolates (Tables 1 and 3). All but 1 C. tropicalis isolate was WT for manogepix (WT-UL, 0.06 mg/liter; 99.3% WT) (Table 3). Susceptibility of C. tropicalis isolates to fluconazole and voriconazole were 95.0% and 95.7%, respectively, according to current CLSI breakpoint interpretive criteria.

Manogepix MIC_50_/MIC_90_ values were >8/>8 mg/liter against the 45 C. krusei isolates tested (Table 1). All (100%) C. krusei isolates were susceptible to anidulafungin and micafungin, 97.8% were susceptible to voriconazole, and all were WT to posaconazole (data not shown).

By comparison with the common species of *Candida* noted above, manogepix was more active against C. dubliniensis (MIC_50_/MIC_90_, 0.004/0.008 mg/liter; 100.0% WT), C. lusitaniae (MIC_50_/MIC_90_, 0.03/0.06 mg/liter; 100.0% WT), and C. auris (MIC_50_/MIC_90_, 0.004/0.015 mg/liter) isolates and less active against C. kefyr (MIC_50_/MIC_90_, 0.25/1 mg/liter; 100.0% WT) isolates (Tables 1 and 3). All isolates of C. dubliniensis and C. lusitaniae were classified as WT to anidulafungin (CLSI ECV, 0.12 and 1 mg/liter, respectively) and micafungin (CLSI ECV, 0.12 and 0.5 mg/liter, respectively) (Table 3). One (6.2%) C. lusitaniae isolate and no (0.0%) C. dubliniensis isolates were NWT to fluconazole (Table 3). The 11 C. auris isolates consisted of 3 isolates from the United States (2 from New York and 1 from Texas), 5 isolates from Greece, and 3 isolates from Latin America (Panama). All C. auris isolates were inhibited by ≤0.03 mg/liter manogepix, and all were susceptible to anidulafungin and micafungin using the CDC tentative MIC breakpoints (Table 3). Of these 11 C. auris isolates, the isolates from New York and Greece were fluconazole resistant, and those obtained from Panama and Texas were fluconazole susceptible.

All 27 C. neoformans var. *grubii* isolates were inhibited by ≤2 mg/liter manogepix (MIC_50_/MIC_90_, 0.5/1 mg/liter) (Tables 1 and 3). In addition, 100.0% of C. neoformans var. *grubii* isolates displayed WT MIC values for voriconazole, fluconazole, and posaconazole (Table 3). Given that echinocandins are commonly utilized for empirical therapy, it is notable that Cryptococcus spp. were intrinsically resistant to this class of agents (Table 3).

### *In vitro* activity of manogepix and comparators against Aspergillus spp. and *Scedosporium* spp. isolates and determination of the wild-type manogepix MIC distribution against Aspergillus spp.

The most common Aspergillus species (containing 10 or more overall isolates) in the 2020 surveillance program that were tested against manogepix included the following four Aspergillus species complexes, in order of frequency: A. fumigatus, Aspergillus section Flavi, Aspergillus section Nigri, and Aspergillus section Terrei. The frequency and cumulative percent inhibition data for manogepix minimal effective concentration (MEC) values against Aspergillus spp. are presented in Tables 1 and 2.

Manogepix exhibited potent *in vitro* activity against all 4 Aspergillus species complexes shown in Table 1, with MEC_90_ values of 0.015 to 0.06 mg/liter. The WT-UL for each species was 0.03 mg/liter for Aspergillus section Nigri (100.0% WT) and Aspergillus section Terrei (100.0% WT) and 0.06 mg/liter for both A. fumigatus (100.0% WT) and Aspergillus section Flavi (100.0% WT) (Tables 1 and 2). All (100.0%) of the Aspergillus spp. tested exhibited a WT manogepix phenotype (WT-UL, ≤0.06 mg/liter) (Table 1).

Manogepix (MEC_50_/MEC_90_, 0.015/0.03 mg/liter) and the echinocandin comparators anidulafungin and micafungin inhibited all 169 A. fumigatus isolates at ≤0.06 mg/liter (Table 5). These isolates displayed WT MEC/MIC results of 100.0%, 87.0%, and 96.4% for manogepix, itraconazole, and voriconazole (91.1% susceptible), respectively (Table 5). Of A. fumigatus isolates, 95.9% were inhibited by ≤0.5 mg/liter posaconazole (MIC_50_/MIC_90_, 0.25/0.5 mg/liter) (Table 5). Twenty-two isolates (13.0%) were NWT to itraconazole; 6 of these isolates were also NWT to voriconazole (MIC, ≥2 mg/liter). Of the 22 A. fumigatus isolates that displayed itraconazole MIC values greater than the CLSI ECV, 11 harbored *cyp*51A or *cyp*51B alterations (Table 6). The most common substitutions were *cyp*51A TR34/L98H (3 isolates) and *cyp*51B Q42L (3 isolates). Four A. fumigatus isolates harbored alterations in *cyp*51A, including 2 isolates with substitution I242V and 1 isolate each with substitutions N248K and G138C. Finally, 1 isolate carried multiple *cyp*51A alterations (F46Y, M172V, N248T, D255E, and E427K) (Table 6). The role of the less frequent alterations in *cyp*51 in clinical resistance to the azoles is unclear, as several have been detected in azole-susceptible isolates. Among the itraconazole NWT isolates with *CYP* alterations, 6 were from the United States (10.2% of North American A. fumigatus isolates), 4 were from Europe (4.3% of European A. fumigatus isolates), and 1 was from the Asia-Western Pacific region (6.3% of Asia-Western Pacific isolates) (Table 6). The manogepix MEC values against the 11 isolates harboring alterations in *cyp*51A or *cyp*51B ranged from 0.008 to 0.06 mg/liter (all <WT-UL) (Table 6).

**TABLE 5:** *In vitro* activities of manogepix and comparator antifungal agents tested against Aspergillus spp. and *Scedosporium* spp.

Organism (no. tested) and antifungal agent	MIC data (mg/liter)			CLSI CBP^*a*^			CLSI ECV^*b*^	
	MIC_50_ or MEC_50_	MIC_90_ or MEC_90_	Range	% S	% I/SDD	% R	% WT	% NWT
A. fumigatus (169)								
Manogepix	0.015	0.03	0.008–0.06				100.0	0.0
Anidulafungin	0.015	0.06	0.004–0.06					
Micafungin	0.008	0.015	≤0.002–0.03					
Itraconazole	1	2	0.25–>8				87.0	13.0
Posaconazole	0.25	0.5	0.06–8					
Voriconazole	0.5	0.5	0.12–8	91.1	5.3	3.6	96.4	3.6
Amphotericin B	1	2	0.5–4				98.8	1.2
Aspergillus section Flavi (24)^*c*^								
Manogepix	0.015	0.06	0.008–0.06				100.0	0.0
Anidulafungin	0.008	0.015	0.004–0.015					
Micafungin	0.008	0.015	≤0.002–0.015					
Itraconazole	0.5	1	0.5–1				100.0	0.0
Posaconazole	0.5	0.5	0.25–0.5				100.0	0.0
Voriconazole	0.5	1	0.25–1				100.0	0.0
Amphotericin B	2	2	1–>4				95.8	4.2
Aspergillus section Nigri (27)^*d*^								
Manogepix	0.008	0.015	0.004–0.03				100.0	0.0
Anidulafungin	0.008	0.015	0.004–0.03					
Micafungin	0.008	0.03	≤0.002–0.03					
Itraconazole	1	4	1–8				96.3	3.7
Posaconazole	0.5	1	0.25–1				100.0	0.0
Voriconazole	0.5	2	0.5–2				100.0	0.0
Amphotericin B	0.5	1	0.5–2				100.0	0.0
Aspergillus section Terrei (14)^*e*^								
Manogepix	0.008	0.015	0.004–0.03				100.0	0.0
Anidulafungin	0.015	0.03	≤0.002–0.06					
Micafungin	0.008	0.015	0.004–0.015					
Itraconazole	0.5	1	0.25–1				100.0	0.0
Posaconazole	0.25	0.5	0.12–0.5				100.0	0.0
Voriconazole	0.5	0.5	0.12–1				100.0	0.0
Amphotericin B	2	4	1–4				100.0	0.0
*Scedosporium* spp. (12)^*f*^								
Manogepix	0.06	0.12	0.03–0.5					
Anidulafungin	4	>4	4–>4					
Micafungin	0.5	>4	0.25–>4					
Itraconazole	>8	>8	2–>8					
Posaconazole	>8	>8	1–>8					
Voriconazole	1	1	0.25–1					
Amphotericin B	>4	>4	1–>4					

a CLSI breakpoint criteria. Susceptible (S), intermediate/susceptible dose-dependent (I/SDD), resistant (R).

b ECV, epidemiological cutoff value; WT, wild type; NWT, non-wild type. The WT-UL was used in place of ECV for manogepix (see Table 2).

c Organisms (number of isolates) included Aspergillus flavus species complex (22) and *A. parasiticus* (2).

d Organisms (number of isolates) included Aspergillus niger (13) and A. niger species complex (14).

e Organisms (number of isolates) included Aspergillus *hortai* (1), A. terreus (6), and A. terreus species complex (7).

f Organisms (number of isolates) included Scedosporium apiospermum*/*Scedosporium boydii (9) and S. aurantiacum (3).

**TABLE 6:** Summary of CYP alterations detected among non-wild-type Aspergillus spp. isolates in the 2020 international surveillance program

Country	Organism	MIC data (mg/liter)^*a*^				CYP alteration(s)	
		Manogepix	Voriconazole	Itraconazole	Posaconazole	CYP51A	CYP51B
USA	A. fumigatus	0.015	0.5 (WT)	2 (NWT)	0.25 (WT)	I242V	WT
USA	A. fumigatus	0.06	1 (WT)	2 (NWT)	0.5 (WT)	WT	Q42L
USA	A. fumigatus	0.015	0.5 (WT)	2 (NWT)	0.25 (WT)	WT	Q42L
USA	A. fumigatus	0.03	0.5 (WT)	2 (NWT)	0.5 (WT)	N248K	WT
USA	A. fumigatus	0.015	0.5 (WT)	2 (NWT)	0.5 (WT)	F46Y, M172V, N248T, D255E, E427K	WT
France	A. fumigatus	0.06	1 (WT)	4 (NWT)	1 (NWT)	WT	Q42L
New Zealand	A. fumigatus	0.03	8 (NWT)	>8 (NWT)	8 (NWT)	G138C	WT
UK	A. fumigatus	0.015	2 (NWT)	4 (NWT)	1 (NWT)	L98H, TR34	WT
UK	A. fumigatus	0.008	2 (NWT)	4 (NWT)	0.5 (WT)	L98H, TR34	WT
UK	A. fumigatus	0.008	2 (NWT)	8 (NWT)	1 (NWT)	L98H, TR34	WT
USA	A. fumigatus	0.015	0.5 (WT)	2 (NWT)	0.25 (WT)	I242V	WT

a Categorical interpretations of non-wild type (NWT) and wild type (WT) are according to ECVs from CLSI document M57 (24). The ECV for posaconazole was 0.5 mg/liter.

Manogepix (MEC_50_/MEC_90_, 0.015/0.06 mg/liter) inhibited all 24 Aspergillus section *Flavi* isolates at ≤0.06 mg/liter (100.0% WT) (Tables 1 and 5) and displayed similar activity as micafungin (MEC_50_/MEC_90_, 0.008/0.015 mg/liter) and anidulafungin (MEC_50_/MEC_90_, 0.008/0.015 mg/liter) (Table 5). All (100.0%) Aspergillus section Flavi isolates were WT to the mold-active azoles (Table 5).

Manogepix (MEC_50_/MEC_90_, 0.008/0.015 mg/liter) inhibited all 27 Aspergillus section Nigri isolates at ≤0.03 mg/liter (100.0% WT) (Tables 1 and 5) and displayed similar activity to micafungin (MEC_50_/MEC_90_, 0.008/0.03 mg/liter) and anidulafungin (MEC_50_/MEC_90_, 0.008/0.015 mg/liter). All but one Aspergillus section Nigri isolate was WT (96.3%) to the mold-active azoles (Table 5).

Manogepix (MEC_50_/MEC_90_, 0.008/0.015 mg/liter) inhibited all 14 Aspergillus section Terrei isolates at ≤0.03 mg/liter (100.0% WT) (Tables 1 and 5). This compound displayed similar activity to micafungin (MEC_50_/MEC_90_, 0.008/0.015 mg/liter) and anidulafungin (MEC_50_/MEC_90_, 0.015/0.03 mg/liter). All (100.0%) Aspergillus section Terrei isolates were WT to the mold-active azoles (Table 5).

Manogepix (MEC_50_/MEC_90_, 0.06/0.12 mg/liter; 100.0% inhibited at ≤0.5 mg/liter) was the most potent compound tested against a collection of 12 *Scedosporium* spp. isolates (Table 5). Corresponding echinocandin (anidulafungin and micafungin) and azole (itraconazole, posaconazole, and voriconazole) MIC_50_/MIC_90_ values were 4 to >4/>4 mg/liter and 1 to >8/1 to >8 mg/liter, respectively (Table 5).

### *In vitro* activities of manogepix against rare species of *Candida*, non-*Candida* yeasts, and rare molds.

Manogepix MIC and MEC values obtained for 24 other *Candida* spp., 26 other yeasts, 12 other Aspergillus spp., and 52 other mold isolates are listed in Table 1 and Tables S1 and S2. Manogepix was active against many uncommon *Candida* spp. isolates, including Candida bracarensis (MIC range, 0.004 to 0.008 mg/liter), Candida duobushaemulonii (MIC, ≤0.002 mg/liter), Candida fermentati (MIC, 0.03 mg/liter), Candida guilliermondii (MIC range, 0.004 to 0.015 mg/liter), Candida haemulonii (MIC, ≤0.002 mg/liter), Candida nivariensis (MIC, 0.004 mg/liter), Candida pelliculosa (MIC, ≤0.002 mg/liter), Candida rugosa (MIC range, 0.008 to 0.03 mg/liter), Candida spencermartinsiae (MIC, 0.008 mg/liter), and Candida utilis (MIC, ≤0.002 mg/liter) (Table S1). Manogepix was also active against infrequently encountered non-*Candida* yeasts, including *Saprochaete clavata* (MIC range, 0.03 to 0.06 mg/liter), Kodamaea ohmeri (MIC, 0.008 mg/liter), Magnusiomyces capitatus (Saprochaete capitata; MIC, 0.015 mg/liter), Rhodotorula mucilaginosa (MIC range, 0.03 to 0.06 mg/liter), Saccharomyces cerevisiae (MIC range, 0.008 to 0.015 mg/liter), and Yarrowa lipolytica (MIC, 0.03 mg/liter) (Table S1).

Notably, manogepix was active against many less common and frequently antifungal-resistant fungi (to azole and/or echinocandin), including Aspergillus nidulans (MEC range, 0.008 to 0.015 mg/liter), Aspergillus sclerotiorum (MEC, 0.015 mg/liter), Aspergillus ustus species complex (MEC, 0.008 mg/liter), Aspergillus versicolor (MEC, 0.015 mg/liter), Exophiala dermatitidis (MEC, ≤0.002 mg/liter), Fusarium incarnatum-equiseti species complex (MEC, ≤0.002 mg/liter), Fusarium solani species complex (MEC range, 0.004 to 0.015 mg/liter), Gibberella fujikuroi species complex (MEC, 0.008 to 0.015 mg/liter), Lomentospora prolificans (MEC range, 0.03 to 0.06 mg/liter), Purpureocillium lilacinum (MEC range, ≤0.002 to 0.008 mg/liter), Paecilomyces variotii (MEC range, 0.004 to 0.008 mg/liter), Penicillium citrinum (MEC, 0.008 mg/liter), Penicillium onobense (MEC, 0.008 mg/liter), Rasamsonia argillacea species complex (MEC range, ≤0.002 to 0.004 mg/liter), and Scopulariopsis brevicaulis (MEC, 0.008 mg/liter). Fungal species with increased MICs to manogepix included Candida inconspicua (MIC range, 0.5 to 2 mg/liter), *Cunninghamella* sp. (MEC, 8 mg/liter), Lichtheimia corymbifera (MEC, 4 mg/liter), *Lichtheimia* sp. (MEC, 4 mg/liter), Mucor circinelloides (MEC range, 0.25 to 1 mg/liter), Mucor indicus (MEC, 1 mg/liter), Rhizopus microsporus group (MEC range, 2 to >8 mg/liter), and Rhizopus oryzae species complex (MEC range, 4 to >8 mg/liter) (Tables S1 and S2).

## DISCUSSION

Recent antifungal surveillance programs have documented the prominent roles of Aspergillus, *Candida*, and Cryptococcus as leading IFI pathogens (1, 2, 12, 20, 25). Although antifungal resistance is a global concern (3, 4), fortunately at present, most clinical isolates of these pathogens remain susceptible or WT to azoles, echinocandins, and polyenes (12). This relatively good news is countered by some less common species of *Candida* and Aspergillus (e.g., C. auris and A. lentulus, respectively), non-*Candida* and non-Cryptococcus yeasts, and non-Aspergillus molds, many of which express intrinsic or acquired resistance to available first-line agents (1–5, 12, 26). Notably, the novel antifungal manogepix exhibits potent antifungal activity against these fungal pathogens (13).

The data presented here expand upon our earlier observations (12, 20) and provide a robust estimate of the WT MIC and MEC distributions of manogepix for 7 species of *Candida* and 4 species of Aspergillus (Table 2). Although multicenter studies involving larger numbers of isolates of each species will be required to establish both ECVs and clinical breakpoints for manogepix, we suggest that the WT-UL values should be ≤0.015 mg/liter for C. dubliniensis (99.4% of 154 isolates), ≤0.03 mg/liter for C. albicans (99.9% of 1,352 isolates) and C. parapsilosis (98.8% of 756 isolates), ≤0.06 mg/liter for C. tropicalis (99.6% of 515 isolates), ≤0.12 mg/liter for C. lusitaniae (97.2% of 107 isolates), ≤0.25 mg/liter for C. glabrata (100.0% of 1,039 isolates), ≤1 mg/liter for C. kefyr (100.0% of 51 isolates), ≤0.03 mg/liter for *A. nigri* (100.0% of 117 isolates) and A. terreus (100.0% of 43 isolates), and ≤0.06 mg/liter for A. fumigatus (100.0% of 748 isolates) and A. flavus SC (100.0% of 115 isolates) (Table 2). These values are comparable to the WT-UL values determined for these species and species groups by the Danish nationwide surveillance program, which reported manogepix species-specific modal MIC values obtained with the EUCAST method (8, 15–17). Thus, both CLSI and EUCAST BMD methods have provided comparable estimates of the *in vitro* activity of manogepix and documented the sustained activity of this agent against yeasts and molds over time.

In the 2020 surveillance program, we confirmed and extended our previous findings regarding the high potency and broad spectrum of manogepix activity against common species of *Candida* and Aspergillus (Tables 1 and 2), as well as against uncommon species of *Candida*, non-*Candida* yeasts, rare species of Aspergillus, and other rare molds (Table 1; see also Tables S1 and S2 in the supplemental material). Given that the major concerns regarding antifungal resistance center on the echinocandins for *Candida* spp. and the triazoles for Aspergillus fumigatus, we utilized whole-genome sequencing to identify *fks* mutations in *Candida* spp. expressing resistance to echinocandins and *cyp*51 mutations in A. fumigatus isolates exhibiting resistance to the mold-active triazoles (Tables 4 and 6). As demonstrated previously, isolates harboring these important resistance mechanisms were all WT for manogepix (8, 12, 15–17, 20). A recent study (27) demonstrated that enhanced efflux expression in Candida albicans and C. parapsilosis mutants was responsible for decreased manogepix susceptibility.

This international surveillance study demonstrated and verified the potent *in vitro* activity of manogepix against contemporary fungal isolates, including echinocandin- and azole-resistant strains of *Candida* and Aspergillus spp. We have expanded the MIC database for manogepix against a broad range of common and uncommon IFI pathogens, and we have shown consistent susceptibility results for manogepix against *Candida* and Aspergillus species over time. The broad spectrum of manogepix is noteworthy for its activity against many less common and often antifungal-resistant yeast and mold strains. Continued development of the manogepix prodrug (fosmanogepix) for the treatment of invasive fungal infections, including multidrug-resistant strains, is warranted.

## MATERIALS AND METHODS

### Organisms.

A total of 1,435 nonduplicate fungal clinical isolates were collected in the SENTRY Surveillance Program during 2020 from 73 medical centers located in North America, Europe, the Asia-Pacific region, and Latin America. The fungal isolates were recovered from patients with bloodstream infections (BSIs; *n *= 693), respiratory tract infections (RTIs; *n *= 253), skin and skin structure infections (SSSIs; *n *= 100), urinary tract infections (UTIs; *n *= 45), intra-abdominal infections (IAIs; *n *= 20), and infections in other sites (*n *= 324).

### Fungal identification methods.

Yeast isolates were subcultured on HardyCHROM agar medium (Hardy Diagnostics, Santa Maria, CA, USA) upon arrival to confirm culture purity for *Candida* spp. isolates and submitted to matrix-assisted laser desorption ionization–time of flight mass spectrometry (MALDI-TOF MS) using the MALDI Biotyper (Bruker Daltonics, Billerica, MA, USA). Any yeast isolates not identified by this process were identified using sequencing-based methods for the internal transcribed spacer (ITS) region, 28S ribosomal subunit, or intergenic spacer 1 for *Trichosporon* spp. (18, 28–30).

Mold isolates were identified by DNA sequencing when an acceptable identification was not achieved by MALDI-TOF MS. For all isolates, 28S was sequenced and 1 of the following genes was analyzed: β-tubulin for Aspergillus spp., translation elongation factor (TEF) for Fusarium spp., or ITSs for all other species of filamentous fungi (18, 28–30).

Nucleotide sequences were analyzed using Lasergene software (DNAStar, Madison, WI, USA) and compared to available sequences using BLAST (https://blast.ncbi.nlm.nih.gov/Blast.cgi). TEF sequences were analyzed using the Fusarium multilocus sequence typing database (https://fusarium.mycobank.org/).

### Susceptibility testing.

Fungal susceptibility testing was conducted according to broth microdilution (BMD) methods as described by Clinical and Laboratory Standards Institute (CLSI) documents M27 (31) and M38 (32). Manogepix MIC and MEC values were determined visually after incubation at 35°C for 24 h (*Candida* spp. MIC) or 48 to 72 h (Aspergillus spp. [48-h MEC], other molds [*Scedosporium* spp. 72-h MEC], other yeasts [48-h MIC], and C. neoformans [72-h MIC]).

Yeast MIC endpoints were read as the lowest drug concentration that produced a significant decrease (≥50% inhibition) of growth below the control for manogepix (23, 31, 33), fluconazole, posaconazole, voriconazole, and the echinocandins, or the concentration preventing any discernible growth for amphotericin B (23, 31). Mold MIC endpoints were read as the lowest drug concentration preventing any discernible growth (amphotericin B, posaconazole, voriconazole, and itraconazole) (32, 34). MEC endpoints (morphology change from flocculent growth to small, matted colonies) were read for manogepix and the echinocandins (18, 32, 34).

Susceptibility interpretive criteria (CBPs and ECVs, where available) were those published in CLSI documents M27 (31), M38 (32), M57S (24), M27M44S (23), and M38M51S (34). Breakpoints for C. auris and amphotericin B, fluconazole, anidulafungin, and micafungin originated from published CDC tentative MIC breakpoints (https://www.cdc.gov/fungal/candida-auris/c-auris-antifungal.html).

CBPs and ECVs have not yet been determined for manogepix against any fungal species. For comparison, previously published manogepix MIC distribution data from the SENTRY surveillance performed in 2017 (20) and 2018–2019 (12) plus the present (2020) survey results were employed to generate a wild-type upper limit (WT-UL; two 2-fold dilutions higher than the modal MIC value of each MIC distribution). This WT-UL was used as the cutoff value to define wild type (MIC ≤ WT-UL) and non-WT (MIC >WT-UL) populations for manogepix and each species (12, 15–17, 20).

Quality control (QC) was conducted according to CLSI documents M27 (31) and M38 (32) using Candida parapsilosis ATCC 22019, Aspergillus flavus ATCC 204304, and Aspergillus fumigatus ATCC MYA-3626. All MIC and MEC values for manogepix against C. parapsilosis ATCC 22019, A. flavus ATCC 204304, and A. fumigatus ATCC MYA-3626 were within QC ranges published in CLSI documents M27M44S (23) and M38M51S (30).

### Resistance mechanisms.

*Candida* spp. isolates showing echinocandin MIC values above the ECV as well as Aspergillus fumigatus isolates displaying azole MIC values above the ECV were subjected to whole-genome sequencing (35). Total genomic DNA was used as input material for library construction prepared using the Illumina DNA library construction protocol and index kit (Illumina, San Diego, CA, USA) following the manufacturer’s instructions. Sequencing was performed on a NextSeq 1000 sequencer (Illumina). Reads were trimmed with Sickle version 1.33 (36) and error corrected using BayesHammer from SPAdes 3.11.1 (37). Each sample was assembled using a reference-guided assembly in DNASTAR SeqMan NGen v.16.0.0 (Madison, WI, USA). DNA regions encoding the FKS hot spots in *Candida* spp. and CYP regions in A. fumigatus were compared to available sequences in the literature.

### Data availability.

Data will be made available upon reasonable request.
