# Supplementary material for: Activities of Manogepix and Comparators against 1,435 Recent Fungal Isolates Collected during an International Surveillance Program (2020)
Source: Antimicrob Agents Chemother. 2022 Oct 26;66(11):e01028-22. doi: 10.1128/aac.01028-22 (PMC9664852; doi:10.1128/aac.01028-22)
Supplement: Supplemental file 1 — Supplemental material. Download aac.01028-22-s0001.pdf, PDF file, 0.2 MB [file aac.01028-22-s0001.pdf]

SUPPLEMENTAL TABLES

Table S1. *In vitro* activity of manogepix and comparators against 50 other yeast isolates

Organism	MIC (mg/L)						
	Manogepix	Anidulafungin	Caspofungin	Micafungin	Fluconazole	Voriconazole	Amphotericin B
Candida bracarensis	0.004	0.12	0.06	0.015	1	0.03	0.5
Candida bracarensis	0.008	0.12	0.06	0.03	2	0.06	0.5
Candida duobushaemulonii	≤0.002	0.12	0.03	0.06	128	0.5	2
Candida fermentati	0.03	1	0.5	0.5	8	0.25	0.5
Candida fermentati	0.03	0.5	0.25	0.25	8	0.12	0.5
Candida guilliermondii	0.004	1	0.25	0.5	2	0.03	0.5
Candida guilliermondii	0.008	2	0.12	0.5	2	0.06	1
Candida guilliermondii	0.015	2	0.25	1	2	0.06	0.5
Candida guilliermondii	0.015	4	0.25	0.5	8	0.12	0.5
Candida guilliermondii	0.004	2	0.5	0.5	2	0.06	0.5
Candida guilliermondii	0.004	2	0.25	0.5	4	0.12	0.5
Candida haemulonii	≤0.002	0.25	0.06	0.12	0.5	0.008	1
Candida inconspicua	0.5	0.03	0.06	0.015	32	0.12	0.25
Candida inconspicua	2	0.03	0.03	0.015	16	0.12	0.5
Candida nivariensis	0.004	0.06	0.03	0.015	4	0.06	1
Candida pelliculosa	≤0.002	0.015	0.015	0.03	2	0.12	1
Candida pelliculosa	≤0.002	0.015	0.03	0.03	4	0.25	0.5
Candida pelliculosa	≤0.002	0.015	0.015	0.03	4	0.12	1
Candida pelliculosa	≤0.002	0.015	0.015	0.06	2	0.12	0.5
Candida rugosa	0.008	0.06	0.12	0.06	8	0.06	0.5

Organism	MIC (mg/L)						
	Manogepix	Anidulafungin	Caspofungin	Micafungin	Fluconazole	Voriconazole	Amphotericin B
Candida rugosa	0.03	0.5	0.25	0.06	2	0.015	1
Candida spencermartinsiae	0.008	0.5	0.12	0.25	4	0.06	0.5
Candida utilis	≤0.002	0.015	0.015	0.015	2	0.12	0.12
Candida utilis	≤0.002	0.03	0.015	0.03	2	0.12	0.5
Cryptococcus gattii species complex	0.25	>4	>4	>4	4	0.06	0.5
Cryptococcus neoformans var. *neoformans*	0.12	>4	>4	>4	1	0.015	0.5
Cryptococcus neoformans var. *neoformans*	1	>4	>4	>4	2	0.03	0.5
Saprochaete clavata	0.06	4	>4	>4	16	0.5	1
Saprochaete clavata	0.03	4	>4	>4	4	0.12	1
Saprochaete clavata	0.03	4	>4	>4	16	0.25	1
Kodamaea ohmeri	0.008	1	0.25	0.5	4	0.03	0.5
Magnusiomyces capitatus	0.015	4	4	>4	1	0.06	1
Rhodotorula mucilaginosa	0.03	>4	>4	>4	32	0.12	0.5
Rhodotorula mucilaginosa	0.06	>4	>4	>4	64	0.5	0.5
Rhodotorula mucilaginosa	0.06	>4	>4	>4	>128	2	0.5
Rhodotorula mucilaginosa	0.03	>4	>4	>4	>128	4	0.5
Rhodotorula mucilaginosa	0.03	>4	>4	>4	>128	2	0.5
Saccharomyces cerevisiae	0.03	0.25	0.12	0.12	16	0.25	0.5
Saccharomyces cerevisiae	0.008	0.12	0.12	0.06	1	0.03	1
Saccharomyces cerevisiae	0.015	0.25	0.25	0.12	4	0.12	0.5
Saccharomyces cerevisiae	0.015	0.12	0.06	0.12	16	0.12	1
Saccharomyces cerevisiae	0.015	0.25	0.06	0.12	4	0.06	0.5

Organism	MIC (mg/L)						
	Manogepix	Anidulafungin	Caspofungin	Micafungin	Fluconazole	Voriconazole	Amphotericin B
Saccharomyces cerevisiae	0.008	0.12	0.06	0.12	8	0.12	0.5
Saccharomyces cerevisiae	0.015	0.5	0.12	0.12	2	0.06	0.5
Trichosporon asahii	>8	>4	>4	>4	4	0.12	1
Trichosporon asahii	0.5	>4	>4	>4	1	0.015	1
Trichosporon asahii	>8	>4	>4	>4	16	0.12	0.5
Trichosporon asahii	>8	>4	>4	>4	128	2	0.5
Trichosporon mycotoxinivorans	>8	>4	>4	>4	8	0.25	2
Yarrowia lipolytica	0.03	0.25	0.12	0.5	2	0.03	1

Table S2. *In vitro* activity of manogepix and comparators against 63 other mold isolates

Organism	MIC or MEC (mg/L)						
	**Manogepix**	**Anidulafungin**	**Caspofungin**	**Micafungin**	**Posaconazole**	**Voriconazole**	**Amphotericin B**
Aspergillus nidulans	0.015	0.015	0.008	0.004	0.25	0.12	2
Aspergillus nidulans	0.015	0.03	0.015	0.004	0.25	0.12	2
Aspergillus nidulans	0.03	0.015	0.03	0.015	0.5	0.12	2
Aspergillus nidulans	0.015	0.015	0.008	0.004	0.25	0.12	2
Aspergillus nidulans	0.008	0.015	0.015	0.004	0.12	0.12	2
Aspergillus nidulans	0.015	0.03	0.015	0.008	0.25	0.12	2
Aspergillus nidulans species complex	0.015	0.008	0.008	0.004	0.25	0.12	2
Aspergillus nidulans species complex	0.015	0.015	0.008	0.004	0.25	0.12	1
Aspergillus sclerotiorum	0.015	0.03	0.015	0.03	0.5	0.5	>4
Aspergillus ustus	0.008	0.03	0.015	0.015	2	4	1
Aspergillus ustus species complex	0.008	0.03	0.015	0.008	>8	8	4
Aspergillus versicolor	0.015	0.015	0.015	0.008	0.25	0.25	1
Exophiala dermatitidis	≤0.002	0.12	0.06	0.015	0.25	0.12	1
Fusarium incarnatum-equiseti species complex	≤0.002	4	1	2	2	2	1
Fusarium solani	0.015	>4	>4	>4	>8	>8	2
Fusarium solani	0.004	0.004	0.008	0.008	>8	8	4
Fusarium solani species complex	0.015	>4	>4	4	>8	8	1

Organism	MIC or MEC (mg/L)						
	Manogepix	Anidulafungin	Caspofungin	Micafungin	Posaconazole	Voriconazole	Amphotericin B
Fusarium solani species complex	0.008	>4	>4	>4	>8	8	2
Fusarium solani species complex	0.015	>4	>4	>4	>8	8	1
Fusarium solani species complex	0.015	>4	>4	4	>8	4	1
Fusarium solani species complex	0.015	>4	>4	>4	>8	>8	2
Gibberella fujikuroi species complex	0.008	0.008	0.008	0.004	2	4	2
Gibberella fujikuroi species complex	0.008	>4	>4	>4	>8	>8	4
Gibberella fujikuroi species complex	0.008	>4	>4	>4	>8	8	2
Gibberella fujikuroi species complex	0.008	>4	>4	>4	>8	>8	2
Gibberella fujikuroi species complex	0.015	>4	>4	>4	1	2	2
Gibberella fujikuroi species complex	0.015	>4	>4	>4	>8	4	2
Lichtheimia corymbifera	4	>4	>4	>4	0.5	>8	0.5
Lomentospora prolificans	0.06	>4	>4	>4	>8	>8	>4
Lomentospora prolificans	0.03	>4	>4	>4	>8	>8	>4
Lomentospora prolificans	0.06	>4	>4	>4	>8	>8	>4
Lomentospora prolificans	0.06	>4	>4	>4	>8	>8	4
Mucor circinelloides	1	>4	>4	>4	2	>8	0.5
Mucor circinelloides	0.25	>4	>4	>4	>8	>8	0.5

Organism	MIC or MEC (mg/L)						
	Manogepix	Anidulafungin	Caspofungin	Micafungin	Posaconazole	Voriconazole	Amphotericin B
Mucor indicus	1	>4	>4	>4	1	>8	0.5
Purpuriocillium lilacinum	≤0.002	0.03	0.03	0.015	0.25	0.06	2
Purpuriocillium lilacinum	0.008	0.06	0.03	0.03	0.5	0.25	>4
Paecilomyces variotii	0.008	0.008	0.008	0.004	0.25	4	0.25
Paecilomyces variotii	0.004	0.008	1	0.004	0.5	8	0.25
Paecilomyces variotii	0.004	0.008	0.008	0.004	0.25	2	0.12
Paecilomyces variotii	0.004	0.008	1	0.004	0.25	4	0.12
Paecilomyces variotii	0.004	0.008	1	0.004	0.25	4	0.12
Paecilomyces variotii	0.004	0.008	1	0.008	0.5	4	0.25
Paecilomyces variotii	0.004	0.008	0.015	0.004	0.25	4	0.12
Penicillium citrinum	0.008	0.015	0.008	0.004	1	>8	1
Penicillium onobense	0.008	0.008	0.015	0.015	0.25	0.5	1
Rasamsonia argillacea	0.004	0.008	0.008	0.008	2	>8	2
Rasamsonia argillacea species complex	≤0.002	0.015	0.015	0.008	0.5	8	1
Rasamsonia argillacea species complex	≤0.002	0.03	0.015	0.015	1	>8	1
Rasamsonia argillacea species complex	0.004	0.008	0.008	0.004	0.5	>8	0.5
Rhizopus microsporus group	2	>4	>4	>4	0.5	8	0.5
Rhizopus microsporus group	2	>4	>4	>4	0.5	8	1

Organism	MIC or MEC (mg/L)						
	Manogepix	Anidulafungin	Caspofungin	Micafungin	Posaconazole	Voriconazole	Amphotericin B
Rhizopus microsporus group	>8	>4	>4	>4	1	8	1
Rhizopus microsporus group	>8	>4	>4	>4	0.5	8	1
Rhizopus oryzae	4	>4	>4	>4	0.5	8	0.5
Rhizopus oryzae species complex	>8	>4	>4	>4	0.5	>8	0.5
Scopulariopsis brevicaulis	0.008	0.5	0.06	0.06	>8	>8	4
Unspeciated *Acremonium*	0.25	0.5	0.12	0.12	>8	8	2
Unspeciated *Coprinellus*	0.008	4	>4	>4	0.25	0.015	0.12
Unspeciated *Cunninghamella*	8	>4	>4	>4	1	>8	2
Unspeciated *Lichtheimia*	4	>4	>4	>4	1	>8	0.5
Unspeciated *Paecilomyces*	≤0.002	0.008	0.008	0.004	0.5	>8	0.5
Unspeciated *Trichoderma*	0.03	0.12	0.015	0.015	1	1	2
